# The effect of probiotic-fortified kefir on cardiovascular risk factors in elderly population: a double-blind, randomized, placebo-controlled clinical trial

**DOI:** 10.1186/s40795-024-00875-5

**Published:** 2024-05-13

**Authors:** Mehran Noori, Zainab Shateri, Siavash Babajafari, Mohammad Hadi Eskandari, Karim Parastouei, Mohammad Ghasemi, Hoseein Afshari, Mohammad Samadi

**Affiliations:** 1 Health Research Center, Life Style Institute, Baqiyatallah University of Medical Sciences, Tehran, Iran; 2 Department of Nutrition and Biochemistry, School of Medicine, Ilam University of Medical Sciences, Ilam, Iran; 3 Nutrition Research Center, Department of Clinical Nutrition, School of Nutrition and Food Science, Shiraz University of Medical Sciences, Shiraz, Iran; 4 Department of Food Science and Technology, School of Agriculture, Shiraz University, Shiraz, Iran; 5 Student Research Committee, Baqiyatallah University of Medical Sciences, Tehran, Iran; 6 Exercise Physiology Research Center, Life Style Institute, Baqiyatallah University of Medical Sciences, Tehran, Iran

**Keywords:** Kefir, Probiotics, *Bifidobacterium*, *Lactobacillus*, Lipid profile

## Abstract

**Introduction:**

The outbreak of cardiovascular disease (CVD) augments with age. Gut dysbiosis can worsen or initiate systemic disorders such as metabolic diseases and CVDs. Therefore, this research aimed to assess the effect of kefir fortified with *Lactobacillus helveticus R0052* and *Bifidobacterium longum R017* on CVD risk factors in the elderly population. The subjects of this study were selected from the Motahari Clinic in Shiraz, Iran.

**Method:**

This study was a double-blind, randomized, and controlled clinical trial that was conducted on 67 elderly people who were randomly divided into two groups: the fortified kefir group (*n* = 32), which received one bottle of fortified kefir (240 cc), and the placebo group (*n* = 35), which received one bottle of regular kefir for eight weeks. To analyze the data, SPSS software was applied.

**Results:**

After eight weeks, significant differences were seen in atherogenic and Castell’s risk index I between the fortified and regular groups (*p* = 0.048 and *p* = 0.048, respectively). No significant differences were found in Castelli’s risk index II, high-density lipoprotein cholesterol (HDL-C), total cholesterol, triglycerides (TG), non-HDL-C, TG-cholesterol index, and fasting blood sugar by comparing the two groups.

**Conclusion:**

Our investigation demonstrated that fortified kefir with probiotics did not significantly affect lipid profiles. Still, it could significantly affect some indices, including Castelli’s risk index I and atherogenic index. More studies are required to confirm the findings and mechanisms of probiotics’ effect on CVD risk factors.

**Trial number:**

The present registered at the Iranian Registry of Clinical Trials (IRCT20130227012628N3) at 2023-02-21.

## Introduction

Today, due to technological and medical advances, people’s life expectancy has increased, which has led to an increase in the average age of society and an increase in the elderly population [1, 2]. “Elderly” refers to a person aged 65 or older [3]. The outbreak of cardiovascular disease (CVD) risk factors augments with age [4]. CVD is one of the most common causes of death worldwide, estimated to have caused 17.8 million deaths worldwide in 2017 [5, 6]. In 1960, the most common causes of death in Iran were diarrheal and infectious diseases, and in the last few decades, it has shifted to CVDs [6]. The growing epidemic of CVDs may be due to insufficient physical activity, urbanization and industrialization, dietary changes, cultural and socio-economic changes, increased life expectancy, and increased metabolic risk factors [6]. Other risk factors for CVDs include obesity, smoking, diabetes, dyslipidemia, and hypertension [7].

Studies have indicated a relationship between gut microbiota and the function of various host organs. Gut dysbiosis can worsen or initiate systemic disorders such as metabolic diseases and CVDs [8, 9]. This issue has led to an increased interest in using probiotics, whose studies suggest their role in preventing intestinal dysbiosis and maintaining intestinal homeostasis [10–12]. A study on rats indicated that milk products fermented by *Bifidobacterium longum*, a probiotic species, can improve lipid profile [13]. It has also been shown that milk fermented with a species of bacteria called *Lactobacillus helveticus* can effectively reduce blood pressure in animal models [14].

Kefir, which is milk fermented from a combination of yeast and bacteria, is the most widely utilized and has attracted the most attention [15]. Various studies have shown that fermented milk with kefir grains can play a role in lowering blood pressure [16, 17]. Also, a study showed that kefir could help reduce hemoglobin A_1_C (HbA_1_C) and blood sugar in patients with type 2 diabetes mellitus. Further, the mentioned research did not show any significant relationship between kefir consumption and lipid profile [18]. However, another study illustrated that kefir consumption can effectively lower lipid profiles and blood pressure in metabolic syndrome [19].

There are some conflicting studies on the relationship between kefir and CVD risk factors. Also, to our knowledge, there has been no research on kefir fortified with two types of bacteria, *Bifidobacterium longum* and *Lactobacillus helveticus*, in elderly subjects. Therefore, this study aimed to investigate the effect of kefir fortified with *Bifidobacterium longum* and *Lactobacillus helveticus* on CVD risk factors in the elderly population.

## Methods

### Trial design

This research was a parallel, randomized, double-blind, and controlled clinical trial. Using G*Power software with d = 0.75, α = 0.05, and β = 80% based on the previous study [20], the sample size was computed for the total antioxidant capacity [21] variable (unpublished data). Therefore, 29 subjects were needed to participate in each group. Considering 20% removal or violation of protocols, 36 participants were required for each fortified and regular kefir groups. The present study was confirmed by the Baqiyatallah Hospital Ethics Committee (ethical no.: IR.BMSU.BAQ.REC.1401.113) and registered at the Iranian Registry of Clinical Trials (IRCT20130227012628N3) at 2023-02-21 (The secondary data of this clinical trial that registered in Iranian Registry of Clinical Trials were reported in the present study).

A permuted-block randomization was used for participants’ randomization with a fixed block size of four by a computer (2:2 ratio). Allocation and randomization were masked from participants and researchers. A trained assistant randomly allocated subjects to fortified and regular kefir groups. To follow a double-blind design, the kefir manufacturing company was asked to code the bottles. Also, each participant was assigned a code to keep the participants’ information confidential. Each participant completed a checklist of personal characteristics and demographic information. At the beginning of the study, height was measured. Also, weight, waist circumference [22], hip circumference (HC), and body mass index (BMI) (weight (kg)/ height (m)^2^) were measured at the start and the end of the study. Seca Germany device was used to measure weight, and non-stretchable and fixed tape was used to measure participants’ height. Participants were asked not to change their usual diet and physical activity during this study. To assess participants’ physical activity, the International Physical Activity Questionnaire (IPAQ) was utilized [23]. The subjects were classified into three groups based on their physical activity level: high: > 3000 metabolic equivalent of tasks (METs)-minutes/week, moderate: 600–3000 METs-minutes/week, and low: < 600 METs-minutes/week. Questionnaires were completed, and blood samples were taken at the beginning and end of the study (after eight weeks). Also, dietary intake was assessed at the beginning and after eight weeks of intervention by a three-day dietary record. Initially, all food portion sizes were changed to grams [24], and then, energy, micronutrients, and macronutrients were calculated by the Nutritionist IV [25].

To regularly consume kefir, we sent reminder messages to all participants, and all participants had to record their kefir consumption every day. Also, the participants were requested to return to the clinic after two weeks to get the next kefir. In addition, all subjects signed informed consent at the beginning of the research.

### Participants

The subjects of this study were selected from the Motahari Clinic in Shiraz, Iran. The participants were only elderly men over 65 years of age. Medical history, lifestyle, drugs, and social information were completed using a checklist. Also, the participants were asked to consume kefir regularly, and how to consume it was explained to them.

The inclusion criteria of our study were the following items: male gender, age > 65 years, BMI > 25, without any history of diseases such as diabetes, CVDs, digestive disorders, chronic infectious diseases, not consuming antibiotics and probiotics in the last two months and not drinking alcohol and smoking.

The present study’s exclusion criteria included changing diet and medication, taking other probiotic supplements, kefir side effects, and being unwilling to continue the research.

### Intervention

Kefir fortified with probiotics and placebo (kefir without added probiotics (regular kefir)) were prepared by Pegah Company, Fars, Iran. The shape, size, and packaging of the two types of kefir were the same. Each bottle of probiotic-fortified kefir (240 cc) contained *Lactobacillus helveticus R0052* and *Bifidobacterium longum R0175* (dosage 3 × 10^9^ colony-forming unit (CFU) for each) (The starter culture of kefir consisted of *LAF4* and *Kluyveromyces marxianus*). In the placebo group, participants received 240 cc of regular kefir simultaneously. The participants were asked to consume a bottle of kefir daily with lunch or dinner for eight weeks. Subjects who consumed less than 80% of kefir were excluded from our research.

### Outcomes

Measurement of non- high-density lipoprotein cholesterol (non-HDL-C), total cholesterol (TC), triglycerides (TG), HDL-C, fasting blood sugar (FBS), Castelli’s risk index I, atherogenic index, TG-glucose index, and Castelli’s risk index II were the primary outcomes of this study, which were evaluated at the start and the end of the 8th week.

### Blood sample collection

Ten mL of fasting (eight hours) blood samples were taken from all subjects at the beginning and end of the study. Then, blood samples were centrifuged at 3000 revolutions per minute (rpm) speed for 7 min, and for further biochemical assessment in the future, isolated serums were frozen at -76 ^o^ C.

### Laboratory analyses

We used enzymatic kits (Pars Azmoon, Tehran, Iran) for measuring lipid profiles and FBS. Also, some indices were calculated based on the bottom formula:

Castelli’s risk index II: low-density lipoprotein cholesterol (LDL-C) (mg/dL) / HDL-C (mg/dL).

Castelli’s risk index I: TC (mg/dL) / HDL-C (mg/dL).

TG-glucose index: Ln [fasting TG (mg/dL) × fasting plasma glucose (mg/dL) / 2]

Atherogenic index: non-HDL-C (mg/dL) to HDL-C (mg/dL).

Non-HDL-C: TC (mg/dL) - HDL-C (mg/dL).

### Statistical analysis

We used the Mann-Whitney U-test and the independent sample T-test for the two groups’ nutrient and cardiovascular risk factor variables. Also, the Mann-Whitney U-test was used for continuous variables, and the chi-square test assessed the qualitative features of the baseline variables. Moreover, the paired sample T-test and Wilcoxon U-test were used for within-group analysis. In addition, the Kolmogorov-Smirnov test was applied to assess the normality of the variables. To analyze the data, SPSS software was applied. A *p* < 0.05 was considered a level of significance.

## Results

**Fig. 1:**
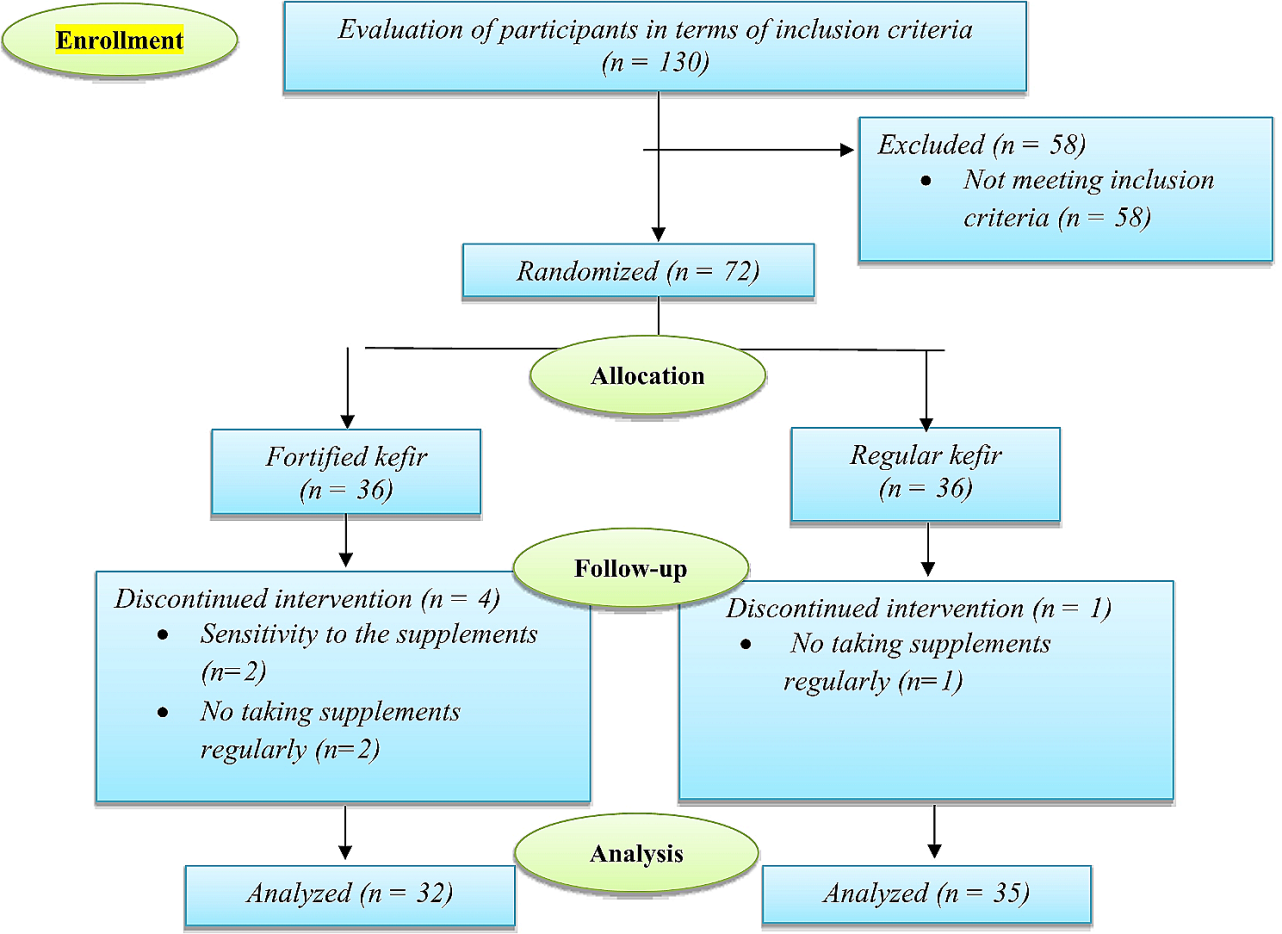
Flowchart of the study

After eight weeks of fortified and regular kefir supplementation, 67 overweight and obese elderly subjects completed the trial (35 people in the regular kefir group and 32 people in the fortified kefir group). The reasons for excluding people are shown in Fig. 1.

The participants’ demographic and anthropometric features of the study are shown in Table 1. According to this table, the difference in anthropometric and demographic features between the two groups was not significant (p ˃ 0.05 for all).

**Table 1:** Anthropometric and demographic features of the study population at the baseline and end of the study (*n* = 67)

Variables	**Fortified kefir group**	Regular kefir group	*P*-value
	**(n = 32)**	**(n = 35)**	
Age (year) ^1^	65.00 (65.00–66.00)	65.00 (65.00–65.00)	0.375
Weight [40] ^1^			
Before	77.00 (73.00–85.00)	77.00 (72.00–85.00)	0.93
After	76.00 (73.00–84.00)	77.00 (72.00–83.00)	0.895
Height (cm) ^1^	170.00 (165.00-174.50)	170.00 (165.00-173.00)	0.924
BMI (kg/m^2^) ^1^			
Before	26.44 (25.31–28.63)	27.28 (25.30-29.38)	0.935
After	26.10 (25.22–28.05)	26.44 (25.09–28.90)	0.93
Waist circumference (cm) ^1^			
Before	99.00 (97.00-105.75)	99.00 (97.00-107.00)	0.915
After	98.50 (96.00-102.00)	98.00 (97.00-104.00)	0.668
Hip circumference (cm) ^1^			
Before	103.00 (101.00-109.75)	103.00 (100.00-113.00)	0.91
After	100.00 (98.00-106.50)	100.00 (98.00-110.00)	0.905
WHR ^1^			
Before	0.96 (0.96–0.97)	0.96 (0.96–0.97)	0.86
After	0.98 (0.96–0.98)	0.97 (0.97–0.98)	0.93
Physical activity, % ^2^			0.806
Low	19 (59.40)	22 (62.90)	
Moderate	13 (40.60)	13 (37.10)	
Education Level, % ^2^			0.771
Less than diploma	8 (25.00)	7 (20.00)	
Diploma and more	24 (75.00)	28 (80.00)	
Smoking history, % ^2^			0.584
Yes	7 (21.90)	10 (28.60)	
No	25 (78.10)	25 (71.40)	
Disease history, % ^2^			0.787
Yes	10 (31.30)	9 (25.70)	
No	22 (68.80)	26 (74.30)	
Medication, % ^2^			1
Yes	10 (31.30)	11 (31.40)	
No	22 (68.70)	24 (68.60)	

- BMI: body mass index, WHR: waist-to-hip ratio, kg: kilogram, cm: centimeter, kg/m: kilogram / meter^2^

-Using Mann-Whitney U-test for continuous and chi-square test for categorical variables

^1^Values are median (25th -75th)

^2^Values are number (percent)

The study population’s nutrient intake at the start and after eight weeks of kefir supplementation is reported in Table 2. By comparing the two groups, there was no significant difference in macro- and micronutrient intake at the beginning and after eight weeks of kefir supplementation (p ˃ 0.05 for all). Also, within-group analysis showed no significant difference in all nutrient intake in the fortified and regular kefir groups (p ˃ 0.05 for all).

**Table 2:** Nutrient intake of the study population at the baseline and end of the study (*n* = 67)

Variables	Fortified kefir group	Regular kefir group	*P*-value ^3^
	(n = 32)	(n = 35)	
Energy (kcal/day) ^1^			
Before	2087.88 (1857.31-2247.46)	2001.87 (1852.87-2350.85)	0.925
After	2079.64 (1854.90-2243.30)	2012.36 (1851.41-2350.36)	0.87
*P*-value ^4^	0.155	0.441	
Carbohydrate (gr/day) ^2^			
Before	270.04 ± 69.72	268.03 ± 58.23	0.898
After	266.20 ± 62.36	277.46 ± 54.63	0.434
*P*-value ^4^	0.755	0.37	
Protein (gr/day) ^2^			
Before	81.35 ± 22.17	86.96 ± 29.28	0.383
After	266.20 ± 62.36	89.79 ± 28.40	0.44
*P*-value ^4^	0.755	0.682	
Fat (gr/day) ^2^			
Before	81.20 ± 25.00	78.62 ± 20.22	0.642
After	79.20 ± 18.17	75.13 ± 18.64	0.37
*P*-value ^4^	0.721	0.442	
Fiber (gr/day) ^2^			
Before	27.96 ± 13.77	27.36 ± 12.09	0.848
After	25.10 ± 10.09	29.45 ± 15.40	0.18
*P*-value ^4^	0.36	0.468	
Magnesium (mg/day) ^2^			
Before	344.91 ± 123.89	330.19 ± 114.65	0.615
After	298.14 ± 88.97	338.78 ± 147.90	0.183
*P*-value ^4^	0.14	0.779	
Zinc (mg/day) ^1^			
Before	9.33 (6.87–12.04)	11.34 (7.63–13.80)	0.156
After	9.81 (6.54–1226)	9.19 (7.87–11.52)	0.754
*P*-value ^4^	0.695	0.179	
Vitamin C (mg/day) ^1^			
Before	82.89 (26.60-171.62)	145.69 (43.32-205.93)	0.253
After	115.47 (40.71–181.30)	75.28 (41.22-176.31)	0.346
*P*-value ^4^	0.588	0.31	
Vitamin B_6_ (mg/day) ^2^			
Before	1.56 ± 0.59	1.60 ± 0.59	0.694
After	1.86 ± 0.95	1.77 ± 0.95	0.639
*P*-value ^4^	0.198	0.341	
Vitamin B_9_ (mcg/day) ^2^			
Before	331.95 ± 24.20	348.09 ± 24.05	0.639
After	334.87 ± 24.34	390.64 ± 77.78	0.512
*P*-value ^4^	0.936	0.615	

- gr: gram, mg: milligram, mcg: microgram

^1^Values are median (25th -75th)

^2^Values are mean ± SD

^3^Using Mann-Whitney U-test for non-parametric and independent sample T-test for parametric variables

^4^Using Wilcoxon U-test for non-parametric and paired sample T-test for parametric variables

Table 3 shows the effect of fortified and regular kefir on CVD risk factors. At the study’s baseline, there was no significant difference in CVD risk factors between the two groups (p ˃ 0.05 for all). However, after eight weeks, significant differences were seen in atherogenic and Castell’s risk index I between the fortified and regular groups (*p* = 0.048 and *p* = 0.048, respectively). Further, in within-group analysis, a significant reduction was seen in TG (*p* = 0.015), atherogenic (*p* = 0.012), TG-glucose index (*p* = 0.001), Castell’s risk index I (*p* = 0.012), FBS level (*p* = 0.029), and Castell’s risk index II (*p* < 0.001) in the fortified kefir group. In addition, in the regular kefir group, a significant decrease was found in Castell’s risk index II (*p* < 0.001) and FBS level (*p* = 0.011).

**Table 3:** The effect of fortified and regular kefir on cardiovascular risk factors (*n* = 67)

Variables	Fortified kefir group	Regular kefir group	*P*-value ^3^
	(n = 32)	(n = 35)	
TG (mg/dL) ^1^			
Before	174.50 (115.00-217.50)	146.00 (122.00-202.00)	0.555
After	133.00 (104.00-155.75)	174.00 (113.00-225.00)	0.105
*P*-value ^4^	**0.015**	0.857	
TC (mg/dL) ^2^			
Before	159.16 ± 27.00	162.43 ± 42.08	0.709
After	155.25 ± 32.39	161.40 ± 35.69	0.462
*P*-value ^4^	0.529	0.847	
HDL-C (mg/dL) ^2^			
Before	34.00 ± 6.85	34.06 ± 7.01	0.973
After	36.13 ± 10.09	33.80 ± 6.76	0.268
*P*-value ^4^	0.18	0.829	
Non-HDL-C ^2^			
Before	125.15 ± 25.11	128.37 ± 38.52	0.69
After	119.12 ± 26.41	127.60 ± 33.18	0.255
*P*-value ^4^	0.244	0.866	
Atherogenic index ^2^			
Before	3.80 ± 1.01	3.85 ± 1.33	0.853
After	3.40 ± 0.77	3.86 ± 1.07	**0.048**
*P*-value ^4^	**0.012**	0.967	
TG-glucose index ^2^			
Before	9.04 ± 0.58	9.01 ± 0.78	0.844
After	8.68 ± 0.61	8.83 ± 0.52	0.284
*P*-value ^4^	**0.001**	0.155	
Castelli’s risk index I ^2^			
Before	4.80 ± 1.01	4.85 ± 1.33	0.853
After	4.40 ± 0.77	4.86 ± 1.07	**0.048**
*P*-value	**0.012**	0.967	
Castelli’s risk index II ^2^			
Before	3.76 ± 1.01	4.09 ± 1.43	0.295
After	2.56 ± 0.63	2.75 ± 0.71	0.251
*P*-value ^4^	**>0.001**	**>0.001**	
FBS (mg/dL) ^1^			
Before	100.00 (88.25-110.75)	97.00 (89.00-110.00)	0.615
After	86.00 (73.25–99.25)	82.00 (73.00-102.00)	0.797
*P*-value ^4^	**0.029**	**0.011**	

TG: triglycerides, TC: total cholesterol, HDL-C: high-density lipoprotein cholesterol, FBS: fasting blood sugar

^1^Values are median (25th-75th)

^2^Values are mean ± SD

^3^Using Mann-Whitney U-test for non-parametric and independent sample T-test for parametric variables

^4^Using Wilcoxon U-test for non-parametric and paired sample T-test for parametric variables

## Discussion

The present clinical trial evaluated the impact of the fortified kefir with probiotics on CVD risk factors in the elderly population. No significant effect was observed with probiotic-fortified kefir consumption compared to regular kefir on Castelli’s risk index II, non-HDL-C, TC, TG, HDL-C, TG-cholesterol index, and FBS based on the comparison between the two groups. Only Castelli’s risk index I and atherogenic index decreased significantly in the fortified kefir group after the intervention compared to the regular kefir group. However, the TG-glucose index, Castelli’s risk index I, atherogenic index, and TG significantly reduced after the intervention in the fortified-kefir group. Also, FBS and Castelli’s risk index II decreased significantly after intervention in both groups.

The findings of our study revealed that fortified kefir with probiotics had no significant effect on variables such as non-HDL-C, HDL-C, TC, and TG. Various studies have been conducted regarding the impact of probiotics on lipid profile, some of which demonstrated that their consumption could improve the lipid profile [26, 27], and some indicated that their consumption did not affect the lipid profile [28, 29]. A clinical trial study on the impact of probiotics in subjects with non-alcoholic fatty liver disease revealed that probiotic consumption (*Streptococcus thermophilus* and *Lactobacillus bulgaricus*) for three months had no significant effect on HDL-C, LDL-C, TC, and TG [29]. Also, Firouzi. et al. demonstrated that consuming several strains of probiotics, including *B. longum*, in people with type 2 diabetes for 12 weeks had no significant impact on HDL-C, LDL-C, TC, and TG [30].

Studies have shown probiotic foods may reduce serum lipid concentrations [31]. Gilliland et al. found that some strains of *Lactobacillus acidophilus* might reduce cholesterol uptake by binding to cholesterol in the intestinal tract [32]. Also, probiotics can reduce cholesterol by hydrolyzing bile salt and thus deconjugating it and connecting with cholesterol and co-precipitation of cholesterol with deconjugated bile acids [33]. In addition, some probiotics utilize cholesterol in their metabolism [18]. In the current study, both groups received kefir, which contains probiotic living microorganisms, and only in the kefir-fortified group, in addition to other probiotics in kefir, did they also receive two other probiotic strains. The presence of probiotics in kefir, the small sample size and the short duration of the intervention may be the reasons for not observing a significant change in the lipid profile.

The current research findings indicated a significant decrease in Castelli’s risk index I and atherogenic index in the comparison of the two groups. In a randomized clinical trial, a significant decrease in TC/HDL-C ratio was found after supplementation with probiotics (*Lactobacillus fermentum*, *Lactobacillus reuteri*, *Bifidobacterium bifidum*, and *Lactobacillus acidophilus*) for 12 weeks in patients with diabetic nephropathy [34]. Also, another randomized controlled trial illustrated that kefir decreased significantly TC/ HDL-C ratio after eight weeks in obese or overweight premenopausal women [35]. Moreover, Chan et al. found that a multi-strain probiotic mixture decreased atherosclerotic plaque development and inflammation in ApoE^−/−^ mice [36]. Hypercholesterolemia, which refers to elevated blood cholesterol, is a major risk factor for atherosclerosis, which causes stroke and myocardial infarction [37, 38]. Reduction in TC significantly reduces the risk of CVDs [39]. Human probiotic microorganisms that have been well-studied for their cholesterol-lowering impacts in animals and humans are mostly related to the genera *Bifidobacterium* and *Lactobacillus* [40]. The mechanism of probiotics lowering cholesterol is not yet fully understood [41]. However, it has been shown that some probiotics and their metabolites can hinder the synthesis and absorption of cholesterol and cholesterol breakdown [42].

The findings indicated a significant reduction in the fortified-kefir group’s TG-glucose index, Castelli’s risk index I, atherogenic index, and TG. A study by Rosa et al. demonstrated that kefir supplementation could decrease liver TG, plasma TG, and fasting glucose in a metabolic syndrome’s animal model [43]. Also, Mafi et al., in a randomized trial showed probiotic supplements could decrease fasting plasma glucose and TG [34]. Moreover, another research by Zavišić et al. has shown that supplementing mice with metabolic syndrome with probiotics (*Lactobacillus helveticus* and *Lactobacillus rhamnosus*) reduced their serum TG and blood sugar levels [44]. Probiotics can effectively lower blood glucose levels through the inhibition of cytokines and reactive oxygen metabolite production that can destroy beta cells [45]. Also, probiotics can stimulate the production of glucagon-like peptides and insulinotropic polypeptides by acting on the bacteria and thus cause muscle glucose absorption [22]. The probiotics’ mechanism action can also be caused by their effect on the peroxisome proliferator-activated receptor-α (PPAR-α), has a critical role in the transport and oxidation of fatty acids. Also, probiotics can upregulate apolipoprotein, which significantly determines serum TG levels [46].

This research is the first one that investigated the effect of kefir fortified with two particular strains of probiotics on risk factors of CVD in Iranian elderly. Also, the double-blind and placebo-controlled research design was one of the strengths of the current study. However, due to limited financial resources, we could not examine the participants’ microbiomes, and we could not determine whether the consumption of prebiotics caused a change in their composition.

## Conclusions

Our investigation demonstrated that fortified kefir with probiotics did not significantly affect lipid profiles. Still, it could significantly affect some indices, including the Castelli’s risk index I and atherogenic index. More studies are required to confirm the findings and mechanisms of probiotics’ effect on CVD risk factors.

## Data Availability

No datasets were generated or analysed during the current study.
